# A qualitative study of how people with severe mental illness experience living in sheltered housing with a private fully equipped apartment

**DOI:** 10.1186/s12888-016-0888-4

**Published:** 2016-06-06

**Authors:** Eirik Roos, Ottar Bjerkeset, Erik Søndenaa, Dag Øyvind Antonsen, Aslak Steinsbekk

**Affiliations:** Department of Public Health and General Practice, NTNU, Norwegian University of Science and Technology, Trondheim, Norway; Municipality of Trondheim, Trondheim, Norway; Faculty of Health Sciences, Nord University, Bodø, Norway; St. Olavs Hospital, Regional Centre for Child and Youth Mental Health and Child Welfare, Trondheim, Norway; Resource Center for Service User Experience and Service Development (KBT Mid-Norway), Mental Health, Trondheim, Norway

**Keywords:** Community mental health care, Serious mental illness, Sheltered housing, Qualitative study

## Abstract

**Background:**

There are a number of supported housing options for people with severe mental illness (SMI), but limited knowledge about residents’ experiences. The aim of this study was to explore how people with SMI experienced sheltered housing consisting of both a private fully equipped apartment and a shared accommodation room for socializing.

**Methods:**

Fourteen people with SMI living in sheltered housing apartments participated in a qualitative study with semi-structured face to face individual or group interviews.

**Results:**

Residents’ access to the service providers in the sheltered housing, who were seen as both “ordinary people” and skilled to observe symptom changes at an early stage, were major factors for the perception of security. In addition, residents highlighted the possibility of living in a fully equipped apartment, and having access to a shared accommodation room to connect with other residents. Having a fully equipped apartment including their own equipment such as a washing machine was said to help reduce conflicts. Short tenancy agreements made some informants feel insecure. It was also essential to have meaningful daily activities outside the residence to avoid re-hospitalization.

**Conclusions:**

The positive experience was connected to having a fully private equipped apartment including shared accommodation room. The service providers should be aware of the dilemma with in-house support, to make residents feel secure versus increased dependency on service providers.

**Electronic supplementary material:**

The online version of this article (doi:10.1186/s12888-016-0888-4) contains supplementary material, which is available to authorized users.

## Background

In the past decades many countries have initiated extensive mental health care system reforms, and the main goal of these reforms has been to transfer treatment and follow up for individuals with severe mental illness (SMI) from psychiatric hospitals to the community [1]. Deinstitutionalization policies represent a shift in practice for the treatment and support of individuals with SMI [2].

A consequence of this shift is that there is an increased need for services in the community. Beside therapeutic communities, supported independent tenancies, residential care or living with family, there are a number of sheltered or supported housing options for people with severe mental disorders [3–7]. Sheltered, supportive and supported housing are often used as equivalent terms and in this article we have chosen to use “sheltered housing.”

The main goal of sheltered housing is often to support the residents in a rehabilitation process to prevent unnecessary admissions to mental health institutions [8, 9]. However, a review of studies on sheltered housing found that most studies in this area did not give enough details to classify them according to eight housing dimensions for people with SMI; 1) the individual owns the housing or has a lease in his/her own name, 2) housing and service agencies are legally and functionally separate, 3) housing is integrated into the community, 4) housing is affordable (i.e., no more than 40 % of adjusted gross income), 5) services (including medication) offered are voluntary, 6) the individual has choice of the housing and services, 7) services are community-based (there are no live-in staff) and 8) crisis services are available 24 h a day, 7 days a week [10]. Another review of the literature on housing approaches for psychiatric consumers found that the main characteristics of sheltered housing were that residents have their own room within a building complex with shared facilities such as a laundry, dining and living rooms and services provided by in-house staff [11]. Sheltered housing thus represents a housing arrangement in between independent living in the community and a residential institution [12] for people who prefer to live independently, but want the security and availability of assistance and care when needed [13, 14].

It is important to know how sheltered housing can be improved as studies from the Netherlands have found that service users living independently were more likely to feel socially included than residents in sheltered housing [15]. Another qualitative study among 40 US adults with dual disorders living in either supervised or independent housing arrangements [16], found that most of the clients living in independent housing arrangements either interacted only with other clients who were also living there or they kept to themselves. In contrast, nearly all clients in supervised housing reported a sense of community among the other tenants and receiving peer support [16].

There is a range of reviews on the effects of different forms of sheltered housing [3, 5, 11, 17–22]. Some of these have focused on participants’ hospital use before and after housing interventions [21, 22], residential stability [5, 10, 22], preventing homelessness for individuals with mental and substance disorders [19] and among individuals discharged from hospitals [21], and how the residents made sense of their occupational transformations in the context of their everyday life and life history [23]. However, relatively few publications have investigated how people with SMI experience living in sheltered housing [4, 7, 16, 24]. We have only identified one study on how people experience living in private, fully equipped apartments with shared facilities [24], a Swedish qualitative study of 29 users with SMI living in fully equipped apartments or single rooms. Previous studies have shown inconsistency regarding social inclusion among those living in independent housing arrangement versus sheltered housing.

The aim of this study was to explore how people with SMI experience living in sheltered housing consisting of *only* private fully equipped apartments including shared accommodation room.

## Methods

This was a qualitative study with semi-structured individual or group interviews. The data collection was conducted from September to November 2014 and started with the first group interviews, then five individual interviews, then the second group interviews and one individual interview.

### Setting

This study took place in Trondheim, a city in Central Norway with 185,000 inhabitants. The municipality offers different types of services to people with severe mental illness (SMI) such as psychosocial support, training services, aftercare, supported housing with traditional case management and sheltered housing. A total of 140 persons with SMI currently live in sheltered housing owned by the municipality. The sheltered houses are organized as units consisting of one building complex with 7 to 30 one person fully equipped apartments with all amenities such as their own bathroom, kitchen and living room. They also have access to a shared accommodation room where the staff also attended. The residents are offered a 3 year tenancy agreement which has to be renewed at the end of the term. The sheltered housing looks like any other private homes in the area and is placed in different residential neighbourhoods. There are facilities like shops and walking areas nearby, and the distance to city centre is 10–20 min by bus.

Live-in staff (employed by the municipality) attended each unit 24 h a day, 7 days a week (24/7). The main purpose of these facilities is to maximize the personal autonomy of residents and encourage them to do as much as possible for themselves including personal care, shopping, cooking, domestic chores and leisure time activities with the support of the staff as needed. Most residents have daily or weekly meetings with a mental health nurse or a service provider to discuss topics such as how to cope with the psychiatric disease, somatic health, household tasks, and financial issues. All services offered are voluntary, meaning that users can decide whether they want to accept help or not.

### Participants

The aim was to include people living in different sheltered housing units who had a diagnosis of SMI (for example: schizophrenia, schizoaffective disorders or major depression, personality disorders) and who had a level of competency to consent themselves. It was aimed to obtain variation in age, gender and number of years of residence in sheltered housing.

To recruit informants, the first author had meetings with unit managers and team leaders of the sheltered housing to inform them about the study so that they could ask residents with a level of competency to consent themselves to participate. The staff was informed about the need for variation among the informants and were asked to take this into account when recruiting. It was emphasized that participation was voluntary. The team leaders were asked to give this information both verbally and as an information sheet to residents who they thought may be interested in taking part in the study. The team leader at each unit organized meetings between the first author and residents who wanted to be interviewed. The first author gave the residents more information about the study and asked if they would like to participate. Then the residents were asked to sign the consent and they could choose whether they wanted to be interviewed individually or in a group. There were no exclusion criteria.

### Data collection

The individual and group interviews took place in the sheltered housing where the informants lived. The first author took part in all the interviews; the fourth author (co-researcher) took part in seven interviews and the third author in one group interview. The co-researcher has previous experiences as a user of mental health services, and experience in asking questions from a user perspective. The group interviews took place in the shared accommodation room in the unit and the individual interviews were conducted in the informants’ own apartments. The staff was not present at any of the interviews. To increase the sense of security, residents from the same sheltered housing were interviewed together in groups. The interviews were audio taped and transcribed verbatim. The average group interview duration was 45 min (range 42–48 min), and the average individual interview duration was 36 min (range 23–64 min).

An interview guide was used (Additional file 1). The main question was to ask about their experiences with their living arrangement, including follow-up questions about what they were most satisfied or not satisfied with, what kind of services influenced their feeling of security, how they experienced living closely with other residents and about their activities during the day.

### Analysis

The data were analyzed, starting after the first interview, using systematic text condensation in an iterative process [25]. This is an iterative four-step process. All authors started by reading and rereading the transcribed interviews separately with an open mind to obtain a general impression, and identify preliminary overarching themes. In the second step, the transcripts were systematically reviewed line by line by the first author and the units of meaning identified, classified and sorted into themes. During all the phases of analysis, the authors had several meetings to discuss meaning-bearing units, core meanings, subthemes and themes according to the purpose of the study. The findings were first categorized into three main themes; experiences with living arrangements, permanent or short tenancy agreement and relations with the staff, but this was later changed to focus on the overall experience and relationships. The third step was to sort the units of meaning into subgroups and reduce the content to a condensate of artificial quotations, maintaining as far as possible the original terminology used by the participants. In the last step, the contents of each code group were summarized into generalized descriptions and concepts.

The recruitment of participants continued until no new themes emerged. At that point, the material was considered saturated. The analysis was performed by the first author and discussed and negotiated with the co-authors. The analysis was validated with a thorough review of the original transcripts of each interview to make sure they were reflected in the results.

## Result

A total of 14 participants (8 men and 6 women) with SMI were recruited from three different sheltered housing units (Table 1). They had lived in the current unit from 2 months to 12 years and none were employed. The most characteristics of the study entrants are frequent hospitalizations and lengthy hospital stay, poor independent living skills and limited social network before they moved to sheltered housing. The informants were interviewed in two group interviews with four participants in each group and six individual interviews.

**Table 1:** Characteristics of the informants (*n* = 14)

Variable	*N*/Mean(SD)
Male	8
Female	6
Age	48.8 years (10.4)
Duration of housing	6.1 years (2.9)
In paid work	0
Disability benefit^a^	14

^a^Disability benefit provides secure incomes to those who have a permanently reduced earning capacity due to illness or injury.

### Experiences with the living arrangements

Participants were especially satisfied to have their own private fully equipped apartment, while at the same time having the opportunity to use the shared living room where they could be with other residents or service providers. It was repeatedly emphasized that the main advantage of sheltered housing is the combination of private and shared accommodation and all residents expressed that they felt safe in the sheltered housing.

*“It’s very good because we have our own apartments and a shared accommodation where we can go if we want to see people.”*

They carried out practical chores in their apartment, such as washing floors, cooking or washing clothes. As expected, they said that they could retire to their own flat if they wish to be alone. This was especially valued when they experienced other residents as having antisocial behaviour or being “in their own world.” Some contrasted this with the situation of being an inpatient at a mental health hospital where they did not have the same opportunity to withdraw.

*“I have a key to the apartment, it is 100 percent private.”*

They used the shared accommodation room to meet other residents for social contact. Examples of activities were participating in common meals once a week, playing games and attending organized activities like cookery courses once a month. However, one resident who had lived in the sheltered housing for 6 years said that although it was good to have shared activities, it was important that not everyone has to participate in all the activities as it would be like being in an institution.

*“Initially there were two shared meals and day care three times a week, and a lot of trips with our own minibus. Now there is much less compared with earlier times. For me this is better because I used to feel guilty if I did not take part.”*

When asked about negative experiences with sheltered housing, the most common comment was about whether or not they could stay there as long as they wanted. The reason was that they have a 3 year tenancy agreement which has to be renewed. Some worried whether their tenancy would be extended.

*“If we behave well and pay the rent on time, I think we should get to stay here as long as we want.”*

### Relations between the residents

Several residents talked about the difficulty of keeping friends when they had longer hospital stays. This was given by some as the reason for having a limited social network. Most of the residents said that they had become friends with one or two other residents. This was said to result in both increased social and physical activity. Some also reported that they take part in more activities now than where they previously lived.

*“I've become better acquainted with some residents that I can go hiking with, go to town or seek low-threshold services.”*

The residents said that it was easier to have social relations with the other residents when they were invited by staff to participate in shared activities or when the staff was present in the shared accommodation room because the staff encouraged people to talk or started activities. Typical topics for small talk were everyday activities such as where to go shopping and if anyone wanted to have a walk or topics from the newspaper. They said that they were encouraged by the staff to not talk about disease, but they still did as this was a topic they all could talk about.

*“I have some friends, but most of them are sick too. I met them during my hospital stay. The staff say we should not talk about disease. But it is disease that is the connection between me and my friends, so we talk about it.”*

The residents also gave examples of situations with bickering or conflicts. Some said that quarrels between residents could make them feel unsafe. Examples of situations that could lead to conflicts were when they were together in the shared accommodation room without any kind of activity, especially on weekends as there were fewer staff and fewer activities. Throughout the interviews, it was talked about how they missed having a mini bus at their disposal to go for trips (they previously had a dedicated mini bus).

*“We residents have no problem with being together, but due to a facility* [the mini bus] *having been taken from us, we are together a lot without activities – so there can be some frictions between us.”*

One resident said that she did not always experience a sense of community with other residents, because of different needs and interests. She instead preferred to go to cafés or a concert with friends with whom she had common interests.

### Relationship with the staff

When asked about how they experienced the staff, it was commonly commented that the staff treated the residents with respect and saw them as human beings and not patients. Words like being treated with dignity, as ordinary people and not as a “diagnosis” were used.

*“I am treated with respect and dignity. The staff said they do not care about diagnoses – they care about people.”*

There were variations in how the residents saw the staff. One resident found it difficult to ask for help sometimes because she felt that she complained too much. Another resident disliked it when the service providers sometimes decided which activities she could attend, which made her withdraw from common activities. Yet another talked about how he saw one of the staff as his friend.

*“I got great support from the nurse in the sheltered housing – I've got very good help and support. I see him as a best friend. He is so nice to talk to. He understands me so well. I feel that he builds me up in a way.”*

Another resident had experienced that when the staff contacted different services, then the problem was taken more seriously than if the resident was making the call. Some of the residents had started to get ambulatory services at the sheltered house, e.g. a psychologist who comes to have conversational therapy. This was highly valued as it was experienced to be easier to follow up.

*“The psychologist can visit me and so there are fewer cancellations. So I am able to have conversational therapy even when I feel a little unwell. When I had a psychologist at the psychiatric hospital, I often cancelled because I could not make myself go there when I was depressed. Now they come to me and so there are many fewer cancellations.”*

Looking back on the time prior to acquiring sheltered housing, many residents described having experienced lack of follow-up services and a life with frequent hospitalizations. It was said that the staff were skilled in observing the residents’ problems and offering counselling and practical help at an early stage. They also said that the staff offered methods so they could cope with symptom changes by themselves.

*“I get good help from the service providers and I do not need help from specialist health services. When I feel depressed, it helps to read a book, watch TV – do something to get your mind on something else.”*

Some also said that the combination of ambulatory services and the security provided by the staff had led to fewer hospitalizations after moving into sheltered housing.

*“I have not had any hospital admissions after I moved here, so I feel safe in the residence.”*

### Contact with family and friends

Most residents said that they had a limited social network outside the sheltered housing. One informant reported that he has only one friend in addition to some of the residents.

*“I have few visits. There is only one person I know in addition to some residents. He calls me once a week to take me for a drive or other activities.”*

Some said that they have contact with family members who, for example, visited and helped to pay bills. Others had support persons that they consider as friends.

*“I have a support person with me to visit the cinema, bowling, go-kart and café visits.”*

Mostly the residents meet their friends outside the residence, for example going for a walk, visiting a café or experiencing cultural or entertainment activities. The advantage expressed was to spend time with others beside the residents.

Most residents said they often visited a low-threshold service to meet other users with mental disorders. However, they did not develop close friendships where they for example visited each other. Low-threshold service in this context means services provides by the municipality where neither scheduled appointments nor referral from specialist is needed.

## Discussion

The main finding was that the residents in this study experience a high degree of security and satisfaction living in sheltered housing, on the one hand due to access to service providers and having the opportunity to seek the shared accommodation room for socializing, and on the other hand due to the possibility of withdrawing into their private fully equipped apartment. Having short tenancy agreements made some informants feel insecure. Nearly all the residents said that they could associate with most residents and often consider one or two residents as friends. They experienced little contact with people outside the sheltered housing and thus focused on activities with the other residents.

### Experiences with living arrangements

There seems to be few studies on the experience of living in sheltered housing where some of the residents had their own fully equipped apartment. In the Swedish qualitative study [24], it is obvious that having a private space gives residents a place with no demands from surroundings or other residents [26], having a fully equipped apartment adds other advantages as well. For example, having the possibility to cook or wash clothes without being dependent on the availability of a shared kitchen or washing machine. Furthermore, it can be experienced as recognition that the residents are “normal” since they have their own fully equipped apartment similar to other citizens. The perspective from a Dutch study [15] confirmed these findings, but their study focused on social inclusion and not the housing arrangements.

Although, such a fully equipped apartment costs between 220 000 and 330 000 euro to build. The Norwegian State Housing Bank (NSHB) offer development programs to municipalities such as finance housing through loans and until 40 % grants of the total cost for building sheltered housing. The residents are paying approximately 36 % of their net income toward rent and utilities and this percentage is slightly larger than the recommendations for permanent supportive housing of 30 % [19]. The cost for a fully equipped apartment is obviously more expensive than a single room with shared accommodation room, but it is considered cost-effective in a Norwegian context if the residents can stay there for long periods without frequent hospitalizations.

One challenge of living in sheltered housing is the close proximity to other residents. Typically, as in this study and described by others [27], residents were allocated sheltered housing without having been involved in the choice of residence. However, nearly all the residents said they could associate with most residents and often considered one or two residents as friends. This confirms findings that sharing a common room and facilities give an experience of being part of a community [16, 24], even if it has also been found that some experience that being brought together was a sign of not being like “ordinary people” [24]. Taken together with the resident`s experience of having few friends, even if residents cannot choose whom they live with, the sheltered housing in this study nevertheless functions as an arena for creating friendship and social contact. The most prominent reason for the sense of belonging given by the residents was the common activities organized by the staff which indicates that some conscious efforts on the part of the staff are needed.

In this study, the residents were unanimous that having the shared accommodation room was very important for them. Although some residents talked about not wanting to take part in all activities and sometimes withdrawing to their own apartment, no one expressed any major problems and conflicts. This is contrary to the Swedish study which found that shared facilities such as a laundry, dining and living room were positive, it was also an arena for conflicts [24]. Examples of situations that led to conflicts were having private property in shared rooms, starting to sing, talk, or quarrel at times that demanded relaxation and silence [24]. We have looked closely at our data, but could not confirm this part of the findings from the Swedish study. One reason might be that the residents in our study did not share this type of information with us, for example due to not wanting to say things that would create further conflicts in the future. This is a very likely explanation for not sharing this type of information in group interviews. However, we also conducted individual interviews in part to overcome this problem, but neither those interviewed individually did not share any stories of conflicts connected to the shared accommodation room. Thus, it is likely that conflicts can arise when sharing common facilities, we think that having a fully equipped apartment including their own equipment such as a washing machine, helps reduce the conflicts. Another important factor is that residents are able to choose whether they wish to make use of the shared accommodation room for socializing or take part in activities organized by staff.

### Permanent or short tenancy agreement

Informants reported feeling insecure due to having a tenancy agreement of only 3 years. A previous study [27] found that it is important to offer an unlimited length of tenancy agreement because SMI is a chronic and fluctuating condition that requires stable surroundings throughout one’s lifetime to maintain health. A recent review [19] also found that consumers consistently rated permanent supportive housing models highest and preferred them over time limited forms of care. Furthermore, stable housing circumstances may be a cornerstone of successful treatment, enabling persons with SMI to transfer their focus from merely surviving to seeking growth opportunities such as life skills programs or addictions treatment [28]. Thus, this literature indicates that it would be wise to give the residents permanent tenancies.

However, if the goal of the sheltered housing is to help some residents to become capable of living on their own in a self-contained apartment, giving this group a permanent tenancy might be counterproductive. It has, for example, been found that many clients indicated that their housing preferences have changed over time and some clients related housing preferences to recovery [29], indicating that a permanent agreement should not be an obstacle to further housing moves [29]. Thus there might be a conflict between having a permanent tenancy and aiming for recovery and independent living.

One possible solution is to design a “housing continua” where the residents move from one housing model to another as they progress in their rehabilitation and recovery [10]. Still, it is important to be aware that such changes would remove the resident from the very environment that enabled him or her to recover, cope and thrive.

### Relations with the staff

The residents described the proximity and duration of relationships with the staff as important factors for feeling confident to seek help from them and having a trusting relationship that gave an experience of security and stability. This resonates with guidance on building trust in mental health services which emphasizes confidentiality and continuity [30]. However others have found that residents in sheltered housing can experience relationships with staff as having a lack of recognition, broken agreements, being checked up on, or that the staff tried to influence daily activities [24]. One reason for this difference could be that the sheltered housing in our study seemed to have a very strong focus on individual skills and resources instead of viewing all residents as a homogeneous group [29, 31]. This is substantiated by some of the residents who had lived in the sheltered housing for a long time who talked about a shift from the staff organizing many common activities to today’s situation with only a few organized activities during the week.

Access to staff 24/7 can cause strong bonds and counteract independency because it could be a hindrance to normalization, participation in society, and recovery [29]. It is important to be aware of the relationship between proximity and distance when assistance is to be provided. Furthermore, it has been speculated that sheltered housing might increase dependency on service providers for people with SMI [3]. However, we found that some of the residents felt more independent to manage on their own and less dependent on services from the staff after a few years. This also indicates that having a focus on individual skills and resources and living rather independently in their own fully equipped apartment (for example making dinner for themselves almost every day) could counteract some of the danger of becoming institutionalized and dependent on the staff.

But we also found that some residents experienced that the staff played a major role in their health care needs as they observed changes in the residents’ mental symptoms and initiated therapy before problems were further aggravated. This was said to be a solely positive aspect of living in sheltered housing as they reported, in line with results from previous studies [5, 21, 32], that they had fewer hospitalizations after they moved into sheltered housing. This raises the question of whether they are still heavily dependent on the staff even if there are clear indications of independent living. One explanation is that the staff managed to allow the residents to live their own life while at the same time observing them. One challenge of this approach could be that the residents become overconfident in their ability to live independently. However, some residents also told how the staff had helped them to find methods to cope with symptom changes by themselves and thus becoming less dependent on the staff.

### Strengths and limitations

The study has several strengths. First, one researcher had the main responsibility of all parts of the study and discussed all issues with the other researchers. Second, a co-researcher participated in 7 of 8 interviews and this helped to establish a relaxed atmosphere and an open dialogue. Finally, participants represent variations in age, gender and time in sheltered housing. It was also important that the researchers had different backgrounds and could interpret the findings from different perspectives.

However, some limitations must be kept in mind. As we did not find other studies from sheltered housing with only private fully equipped apartments, these types of sheltered housing might not be a common one. This also implies that the findings might not be representative for other types of sheltered housing in other countries. It could be that the residents did not talk about conflicts and negative experiences due to a fear that others might get to know what they had said to the researchers. Finally, although it was experienced that the interviews went reasonable well, having interviews with people with SMI is challenging for example due to short and repetitive answers. It could have strengthened this study if the data collection also included long-term observation in the sheltered housing and interviewing the staff. However, this was not done, as the aim of the study was to explore the experience of the residents.

## Conclusions

All residents in the study highlighted the importance of access to the service providers and their skills in observing symptoms at an early stage. They also emphasized the shared accommodation room as important to establish a relationship with other residents. The cost for a fully equipped apartment is more expensive than a single room, but the findings in this study indicate that it has many advantages for the residents. Having a short tenancy agreement made some informants feel insecure, but if the goal of the sheltered housing is to help some residents become capable of living in their own self-contained apartment, giving this group a permanent tenancy might be counterproductive. However, further research is required, preferably looking at possible differences in long-term outcomes in sheltered housing with single room compared to fully equipped apartments. The service providers should be aware of the dilemma with in-house support, to make residents feel secure versus increased dependency on service providers.

## Abbreviations

NSHB, Norwegian State Housing Bank; SMI, Severe mental illness, typically people with a diagnosis of schizophrenia, schizoaffective disorders, major depression, or personality disorders

## Additional file

Additional file 1: Interview guide. Questions related to following main themes; Housing, Municipality services and Open Question about other experiences with other types of community services. (DOC 31 kb)
