# Supplementary material for: A qualitative study of how people with severe mental illness experience living in sheltered housing with a private fully equipped apartment
Source: BMC Psychiatry. 2016 Jun 6;16:186. doi: 10.1186/s12888-016-0888-4 (PMC4895881; doi:10.1186/s12888-016-0888-4)
Supplement: Additional file 1: — Interview guide. Questions related to following main themes; Housing, Municipality services and Open Question about other experiences with other types of community services. (DOC 31 kb) [file 12888_2016_888_MOESM1_ESM.doc]

**INTERVIEW GUIDE**

**(**Individual interviews and group interviews with residents living in sheltered housing).

**The purpose** of this study is to explore how people with severe mental illness (SMI) experience living in sheltered housing consisting of only private, fully equipped apartments including shared accommodation room.

**Main themes**

**Housing**

1. Could you please tell me about your present housing? Is it owned by the municipality or is it a private housing?
   - Are you living alone or together with someone?
   - In your residence, is there also a shared accommodation room?
2. What is your experience living in sheltered housing compared with previous housing?
3. What are you most satisfied with regarding these sheltered housing facilities?
4. What are you not satisfied with regarding these sheltered housing facilities?
5. Is there other type of housing that you would rather prefer to live in?

**Municipality services**

1. Could you please tell me about the services the municipality offers?
2. What are you most satisfied with regarding the services the municipality offers?
3. What are you least/not satisfied with regarding the services the municipality offers?
4. Could you please tell me about how you experience safety;
   - In the sheltered housing
   - To other residents
   - Mental health services
   - Your own mental problems

- How do you manage to live with the symptoms?

1. Could you please tell about how you manage everyday life?

- Working
- Leisure activities
- Social contact

1. When you notice symptom change/increase – how do get access to help?

- Help yourself
- Access to staff or others?

1. Do you have a contact person among the municipality staff that is easy to get in touch with?
2. Are there other types of assistance that you miss or would have preferred to what you get now?

**Open question**

Could you please tell if you have experience with other types of community services, other than what we have talked about now?
